# Successful Treatment With Venetoclax Plus Azacytidine Combined With Radiation Therapy and Donor Lymphocyte Infusion in a Patient With Extramedullary Relapse of Acute Myeloid Leukemia After Stem Cell Transplantation

**DOI:** 10.7759/cureus.53655

**Published:** 2024-02-05

**Authors:** Shotaro Shirato, Satoshi Iyama, Akihito Fujimi, Satoshi Takahashi, Masayoshi Kobune

**Affiliations:** 1 Department of Hematology, Sapporo Medical University School of Medicine, Sapporo, JPN; 2 Division of Laboratory Medicine, Sapporo Medical University Hospital, Sapporo, JPN; 3 Department of Hematology, Sapporo Kiyota Hospital, Sapporo, JPN; 4 Department of Infection Control and Laboratory Medicine, Sapporo Medical University School of Medicine, Sapporo, JPN

**Keywords:** donor lymphocyte infusion, radiation therapy, venetoclax, stem cell transplantation, acute myeloid leukemia, extramedullary relapse

## Abstract

Extramedullary (EM) relapse after allogeneic hematopoietic stem cell transplantation (allo-HSCT) for acute myeloid leukemia (AML) is rare and causes systemic relapse. Consequently, the prognosis is very poor because limited treatment is feasible in post-transplant patients. The efficacy and safety of venetoclax (VEN), a newly developed oral inhibitor of B-cell leukemia/lymphoma-2, plus azacytidine (AZA) in patients newly diagnosed with AML who are ineligible for intensive chemotherapy have been reported. We report a case in which VEN + AZA salvage treatment following radiation therapy and donor lymphocyte infusion afforded promising results in a patient with AML who showed post-allo-HSCT EM relapse.

## Introduction

Extramedullary (EM) involvement is noted in 2-9% of patients newly diagnosed with acute myeloid leukemia (AML) [1] and in 5-12% of patients who receive allogeneic hematopoietic stem cell transplantation (allo-HSCT) owing to relapse [2]. Systemic treatment is required because EM relapse always occurs in patients with underlying bone marrow disease [3]. However, many patients with relapsed AML after allo-HSCT are ineligible for intensive chemotherapy because of organ damage due to HSCT-related treatment and/or graft-versus-host disease.

Venetoclax (VEN) is an oral inhibitor of the BH3 domain of B-cell leukemia/lymphoma-2 (Bcl-2), an anti-apoptotic protein. Overexpression of Bcl-2 promotes tumorigenesis in various cancers, including AML [4]. Pan et al. demonstrated that selective, on-target Bcl-2 inhibition using ABT-199 (VEN) is effective in AML [2]. A pivotal phase 3 placebo-controlled trial (VIALE-A trial: NCT02993523) reported the efficacy and safety of VEN plus azacytidine (AZA) compared with placebo plus AZA in patients with newly diagnosed AML who were ineligible for intensive induction therapy [5]. The VEN plus AZA therapy increased overall survival (14.7 and 9.6 months, respectively) and complete remission (CR) and CR with incomplete blood count recovery (CRi) rate (66 and 28%, respectively). In contrast, in a retrospective analysis, relapse after VEN-based therapy was noted in 29 patients with AML who had undergone allo-HSCT [6]. The overall response rate was 38%, with 28% of patients achieving CR/CRi. However, only a few studies have reported the efficacy of VEN-based therapy in the management of EM relapse, particularly after allo-HSCT.

Here, we report a case in which VEN combined with AZA as a salvage treatment following radiation therapy (RT) and donor lymphocyte infusion (DLI) afforded promising results in a patient with AML who showed EM relapse after allo-HSCT.

## Case presentation

**Figure 1:**
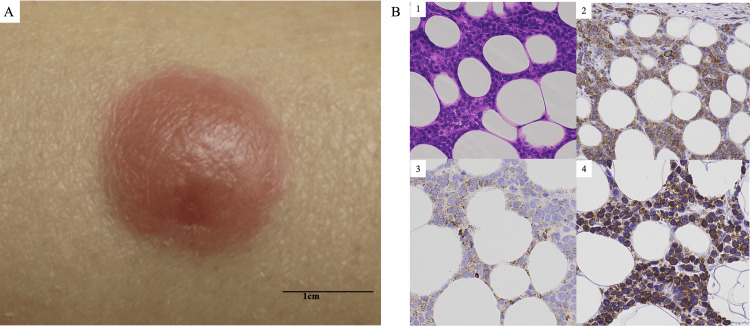
Macroscopic and histological findings of subcutaneous tumors in the right upper arm (A) Macroscopic findings (B) Histological findings: (1) hematoxylin-eosin, (2) CD117, (3) CD34, and (4) BCL-2 staining (200×)

A 61-year-old woman visited a hospital in January 2020 with the chief complaint of low-grade fever and general malaise. She was diagnosed with AML (French-American-British classification M5b) with a normal karyotype and wild-type fms-like tyrosine kinase 3 (FLT3) gene mutation by bone marrow biopsy. After the diagnosis of AML, induction and consolidation chemotherapy were initiated according to the AML201 protocol [7,8], and CR was achieved. After four cycles of consolidation therapy, the patient underwent allogeneic bone marrow transplantation from an HLA-identical and sex-matched unrelated donor after conditioning with fludarabine (30 mg/m^2^ on days −7 to −2), busulfan (3.2 mg/kg on days −5 to −4) in September 2020. We decided on reduced-intensity conditioning because of the patient’s vulnerability. The hematopoietic cell transplantation-specific comorbidity index (HCT-CI) score was 0. Post-transplant GVHD prophylaxis was with 0.03 mg/kg/day of tacrolimus and short-term methotrexate (10 mg/m^2^ intravenously on day 1 and 7 mg/m^2^ intravenously on days 3, 6, and 11). We tapered the dose of tacrolimus and discontinued it in April 2021 (day 215). In June 2021, she was hospitalized for subcutaneous tumors in the right upper arm and both lower legs, without subjective symptoms. A well-circumscribed red nodule 12 mm in diameter and with a smooth surface was observed in the right upper arm (Figure 1A).

**Figure 2:**
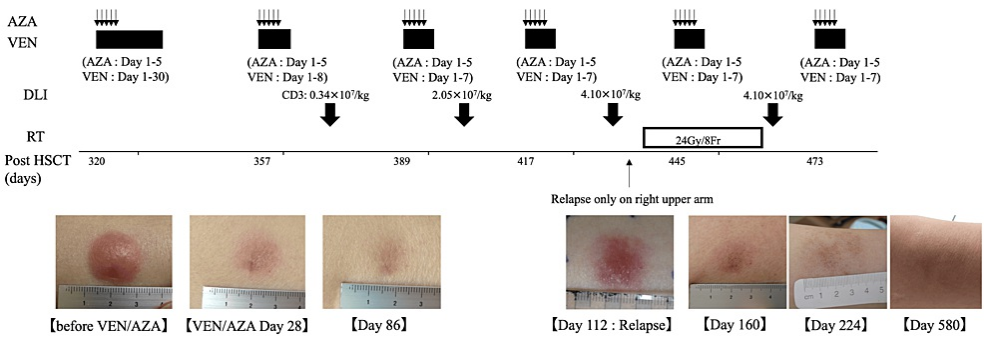
Clinical course VEN, venetoclax (day 1: 100mg; day 2: 200 mg; day 3: 400 mg; later second course: 400 mg); AZA, azacitidine (days 1-5: 75 mg/sqm); DLI, donor lymphocyte infusion; CD3, CD3 positive cells; RT, radiation therapy

On both lower legs, a clearly demarcated region with erythema of approximately 10 mm was observed. The rash on the legs was morphologically similar to that on the arms. Blood tests showed no abnormal findings except mild macrocytic anemia (Hb, 11.2 g/dL). Plain computed tomography showed no abnormal findings, such as hepatosplenomegaly or mass lesions. Bone marrow examination revealed no blast proliferation; chimerism was maintained in 97.4% of donor cells. Hematoxylin-eosin and immunohistochemical staining of the biopsy specimen of the subcutaneous tumor on both the arm and the leg site showed abundant infiltration of atypical cells that were positive for CD34 and CD117 and strongly positive for BCL-2 staining (Figure 1B). The patient was diagnosed with EM relapse of AML. The patient refused intensive chemotherapy following the second stem cell transplantation and received combination chemotherapy with VEN and AZA. AZA was administered for five days at a standard dose (75 mg/m^2^) with a normal dose of VEN in July 2021 (Figure 2).

DLI was started in September 2021. Chemotherapy was continued, while VEN/AZA was suspended to ensure a chemotherapy-free period for infused lymphocytes. The tumors in both the arm and leg sites disappeared completely on day 86 after VEN/AZA therapy. The subcutaneous tumor in the right upper arm regrew in November 2021, and RT (24 Gy/8 fractions) was administered to this site. After six courses of VEN + AZA and four rounds of DLI, the skin lesions were reduced, and there has been no clear recurrence 580 days after the start of treatment. No severe adverse events or graft-versus-host disease development due to VEN/AZA and DLI or irradiation were observed. To determine somatic genomic alterations, we analyzed the genomic profile by next-generation sequencing (NGS) using formalin-fixed paraffin-embedded specimens. We performed genomic profiling using a customized gene panel including 21 genes (DNMT3, FLT3, IDH1, IDH2, JAK2, KRAS, NRAS, PTPN11, SETBP1, SF3B1, SRSF2, U2AF1, ASXL1, BCOR, EZH2, RUNX1, STAG2, TET2, TP53, ZRSR2, and NPM1). No significant mutations affecting VEN/AZA treatment were detected.

## Discussion

High BCL-2 or MCL1 gene expression has been observed in cases of M0 or M1 AML; blast cells have shown high sensitivity to VEN in such cases. However, low BCL-2 or MCL1 gene expression was observed in M4 or M5 AML cells, including monocytic blast cells, which showed resistance to VEN [9]. Bcl-2 protein expression, measured by western blotting, correlated with VEN sensitivity [10]. Overexpression of Bcl-2 was confirmed by immunohistochemistry in our case, which may have resulted in a successful effect regardless of the M5 subtype. In vitro studies have shown that inhibition of BCL-2 may eradicate leukemia stem cells [11]. In contrast, overexpression of other anti-apoptotic proteins, such as MCL-1, has been associated with resistance to chemotherapy in AML [12]. AZA, in a synergistic effect with VEN, induces upregulation of NOXA and reduction of MCL-1 [13,14]. RT is a common treatment for cancer and remains an effective option that can be combined with many anticancer drugs. RT causes DNA strand breaks that increase the activity of pro-apoptotic proteins of the BCL-2 family, such as BAX, NOXA, and PUMA, making RT a rational candidate for combination with BCL-2 inhibitors. In fact, VEN exerts synergistic effects with RT [15]. The graft-versus-leukemia effect and DLI have limited efficacy at EM sites, such as the skin, central nervous system, and gonads [16]. The immune-mediated antitumor effect of RT could lead to the regression of metastatic tumors that are distant from the irradiated field, which is called the “abscopal effect.” The combination of RT and immunotherapy is expected to be effective because immunotherapy can enhance the systemic antitumor effect. Liu et al. reported the abscopal effects of RT combined with an immune checkpoint inhibitor [17].

We administered VEN-based chemotherapy according to the high expression of BCL-2 in EM tumors, followed by a long-term complete response. It is possible that some immune evasion, such as by minor antigens, causes relapse. However, we considered the possibility that leukemia stem cells were eradicated by VEN + AZA therapy with RT and DLI, and no relapse has been observed in this patient to the current date. This work has some limitations. First, we observed only BCL-2 expression levels using immunohistochemistry staining analysis. The use of only the BCL-2 protein assay may have led to an incorrect interpretation of the complex interaction of BCL-2-related proteins, such as BCL-XL and MCL1, which confer resistance to VEN. BH3 profiling is a functional assay that can facilitate the detection of BCL-2 dependence in AML cell lines [10]. Thus, the BH3 profiling technique may be a predictive biomarker for response to VEN treatment in the future. Second, we performed genomic profiling using a customized gene panel including only 21 genes as described above. Comprehensive gene profiling (CGP) may have revealed significant gene mutations in AML in this patient. CGP using NGS is expected to be performed routinely in the near future.

## Conclusions

We report a case in which VEN + AZA salvage treatment following radiation therapy and donor lymphocyte infusion afforded promising results in a patient with AML who showed post-allo-HSCT EM relapse.
